# The Impact of Behavioral Biases on Herding Behavior of Investors in Islamic Financial Products

**DOI:** 10.3389/fpsyg.2020.600570

**Published:** 2021-02-04

**Authors:** Sajid Mohy Ul Din, Shabra Khalid Mehmood, Arfan Shahzad, Israr Ahmad, Alla Davidyants, Ayman Abu-Rumman

**Affiliations:** ^1^School of Economics, Finance, and Banking (SEFB), Universiti Utara Malaysia, Changlun, Malaysia; ^2^Lahore Business School, University of Lahore, Lahore, Pakistan; ^3^Othman Yeop Abdullah Graduate School of Business (OYAGSB), Universiti Utara Malaysia, Changlun, Malaysia; ^4^College of Business (COB), Universiti Utara Malaysia, Changlun, Malaysia; ^5^Department of Propaedeutics of Dental Diseases, I. M. Sechenov First Moscow State Medical University (Sechenov University), Zelenograd, Russia; ^6^Business School, Al-Ahliyya Amman University, Al-Salt, Jordan

**Keywords:** illusion of control, self-attribution bias, Islamic financial products, behavioral biases, herding, Islamic (shari’ah) financial products

## Abstract

The study aimed to investigate the impact of behavioral biases on herding for Islamic financial products with the mediation of shariah literacy. An adopted questionnaire from several published studies was used to collect data. The data were collected from 410 respondents and were analyzed with SmartPLS. The results for the direct impact showed that self-attribution, illusion of control, and information availability have a positive and significant impact on herding for Islamic financial products while shariah literacy showed an insignificant impact on herding. The results for mediation showed that previously significant and positive impact turned to insignificant when shariah literacy was introduced as mediating variable between the illusion of control, self-attribution, information availability, and herding. From a theoretical perspective, this study would contribute to the existing body of knowledge of financial decision making from shariah literacy point-out. On the other hand, the findings of this study may be useful for investors to avoid herding in the Islamic financial markets. The authors synthesize the contribution made by behavioral finance studies in extending the knowledge of herding behavior in Islamic financial products with a mediating role of shariah literacy. The key limitation of the study includes data that were collected from three districts of Punjab, Pakistan.

## Introduction

Traditionally, the efficient market hypothesis (EMH) theory was considered as one of the key foundations for investment decision making. Under EMH, no investor can earn abnormal returns, over and above from average market returns, based on his knowledge and information-processing capabilities (Alnajjar, 2013; Ross et al., 2016). It assumes that investors are rational and utilize a diverse range of models to shortlist and select the optimal investment opportunities. However, EMH failed to justify market anomalies, which resulted in different financial crisis, i.e., internet bubble burst of the 1990s, dot-com crisis, stock market crash of 2002, and bank leading crises of 1994 (Sharma and Kumar, 2019). The opponents of EMH such as Shefrin and Statman (2011), Pompian (2012), and Barberis (2017) argued that investors not necessarily act rationally as claimed in EMH and that their irrationality may be explained through different behavioral anomalies. EMH also ignores the behavior and different mood swings of investors (Aigbovo and Ilaboya, 2019). Past studies reported that several factors such as herd behavior (Babajide and Adetiloye, 2012), psychological factors (Chaffai and Medhioub, 2018), and emotions and cognitive biases (Shefrin and Statman, 2011) were mainly responsible for irrational investor’s behavior. It is thought that investors have more influence on the word of mouth of their family, friends, and peers. Herd behavior in financial markets led to different financial crises, bubbles, and eventually stock crashes (Precher, 2010). For example, the study of Armansyah (2018) found that herd behavior led to financial crisis and stock market crashes in Argentina (2000–2006) and Asia (1997–1998) and the dot-com crash (2008–2009). Following others is much easier than to investigate a matter and then reach the truth.

Low financial literacy may result in information asymmetry and increased herding (Setyowati et al., 2018). Past studies such as Gerardi et al. (2013), Klapper et al. (2013), Lusardi et al. (2014), and Cueva and Rustichini (2015) reported that low financial literacy was the underlying reason for the 2007–2008 global financial crises. Herding creates instability in the market even if it is rational herding. Sharia literacy may also help investors avoid herding, which may subsequently result in fewer market failures (Razak and Abdullah, 2015). Islam encourages critical and rational thinking self-evaluation and discourages herding. In the Quran, Allah said I give men the power of thinking called *aql*. By this fact, a rational person must think critically and analyze the situation by his mind not only to follow the acts of others but also to find out the truth from falsehood. Arabic terms *ya’qilu* (rationalizing) and *yufakkiru* (thinking) are repeated (more than 20 times) in the holy Quran, describing the importance of thinking activity in all actions (Hashim, 2003). God has forbidden herding in several Quranic verses:


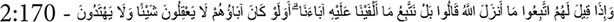


And when it is said to them, “Follow what Allah has revealed,” they say, “Rather, we will follow that which we found our fathers doing (herding).” Even though their fathers understood nothing, nor were they guided?


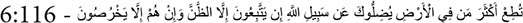


And if you obey most of those upon the earth, they will mislead you from the way of Allah (a crowd may led you to the wrong direction). They follow not accept assumption, and they are not but falsifying.


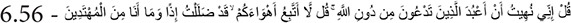


Say, “Indeed, I have been forbidden to worship those you invoke besides Allah.” Say, “I will not follow your desires, for I would then have gone astray, and I would not be of the (rightly) guided.”


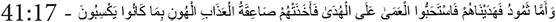


And as for Thamud, We guided them, but they preferred blindness over guidance, so the thunderbolt of humiliating punishment seized them for what they used to earn.


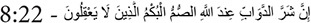


Indeed, the worst of living creatures in the sight of Allah are the deaf and dumb who do not use reason.


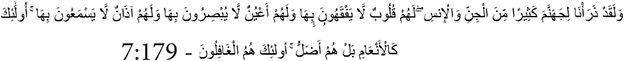


And we have certainly created for Hell many of the jinn and mankind. They have hearts with which they do not understand, they have eyes with which they do not see, and they have ears with which they do not hear. Those are like livestock; rather, they are more astray. It is they who are the heedless.


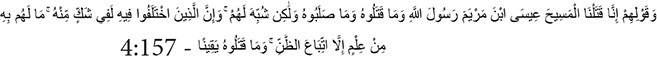


And (for) their saying, “Indeed, we have killed the Messiah, Jesus, the son of Mary, the messenger of Allah.” And they did not kill him, nor did they crucify him; but (another) was made to resemble him to them. And indeed, those who differ over it are in doubt about it. They have no knowledge of it except the following of assumption. And they did not kill him, for certain.


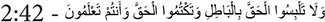


And do not mix the truth with falsehood or conceal the truth while you know (it).


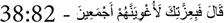


(Iblees) said, “By your might, I will surely mislead them all.”

Previous studies such as Raut (2020), Okello et al. (2017); Alaaraj and Bakri (2020), and Khan (2020) examined investor’s decision making with the mediation of financial literacy for non-Islamic financial products while sharia literacy may have the ability to better explain investor’s behavior. According to the best of the authors’ knowledge, little research has been conducted on sharia literacy for Islamic financial products. Hence, the objective of this study is to explain investor decision-making behavior with the mediating role of sharia financial literacy for Islamic financial products.

## Literature Review

Prospect theory was developed in 1979 by Kahneman and Tversky (1979) who postulated that investors weight losses more than gains, thereby putting higher preference for certain small profits compared with uncertain large profits. Developed another theory for investment decision making as cited by Pompian (2012), i.e., bounded rationality, which assumes that humans have limited information-processing capability, so therefore, they use different heuristics to make decisions (Shefrin and Statman, 2011). Prospect theory coupled with bounded rationality was used to underpin the theoretical model for this research. As applied to our study, these theories hold that we would expect investors be following herding as a means to choose among different Islamic financial products because they have less sharia financial literacy, fewer information availability and processing capabilities, less illusion of control, and self-attribution.

Herding is a common phenomenon in society, and it is defined by Chiang and Zheng (2010) as the habit of following other investor’s actions. Although the classification of herding is not an easy task, various behavior patterns such as imperfect expectations, frequent changes without much new information, bubbles, trends, and mania and sunspot equilibria of an individual can define it. Herding behavior depends on the degree of uncertainty, insufficient knowledge, and less confidence in information processing (Fernández et al., 2011). Kumar and Goyal (2016) defined that herding arise in the market when an investor does not have enough information and does not have certain knowledge to be relied upon for investment decision. Chen et al. (2020) argued that herding tendency was observed because of lack of information or familiarity with the situation and on actions that have been done previously in the same situation. Due to lack of information of the investors involved in the habit of following other investors, they assume that they are better in processing the best option for investment in decision making. Herding not only affects investor’s returns but may also result in market inefficiencies (Arisanti and Oktavendi, 2020). Herding, in the financial markets, is the tendency of investors to follow the investment behavior of others, probably irrationally (Pompian, 2012). It arises when an investor makes the same decision on the investment made by the majority because of lack of information-processing capabilities and uncertainties (Obeng, 2020). Similarly, Fernández et al. (2011) argued that investors who have inadequate information, scarce knowledge, and less confidence in their beliefs are usually involved in herding behavior. However, herding may lead to irrational investment decisions (Chiang et al., 2013).

Sabir et al. (2019) examined the relationship between illusions of control, self-attribution, herding behavior, and information availability on investor’s decision making. This study followed a survey questionnaire approach to collect data from 300 respondents. The authors concluded that illusion of control and self-attribution favor investor’s herding while greater information availability may lead to more logical, reasoned, and rational behavior, discouraging herding. Another study by Abreu and Mendes (2012) examined the impact of information available on the trading behavior of investors. The data for this study were collected through the questionnaire, and the results showed that investors with more information have a higher tendency to trade more frequently. Similarly, a study by Tauni et al. (2015) also reported a positive relationship between information availability and trading frequency for Chinese investors. Investors spend a significant amount of time in finding, collecting, and analyzing information to make prudent, intelligent, and rational investment decisions. Literature such as Precher (2010), Fernández et al. (2011), Chiang et al. (2013), and Sabir et al. (2018) reported that large investors have less herding tendency than small investors because large investors may utilize diverse sources to collect and analyze available information, thereby refraining to indulge in herding, while small investors may simply follow the large investor’s investment behavior.

Arisanti and Oktavendi (2020) investigated the relationship between information type (good governance business shariah disclosure) and herding. The data for this study were collected from the listed companies at Indonesia Stock Exchange, and the results revealed a significant impact of information disclosure on herding. Another study by Tiniç et al. (2020) also examined the relationship between informed trading and herding for Borsa, Turkey, investors. This study found a significant relationship between informed trading and herding, and the effect of herding would be stronger in the case of short-selling restriction. The findings of this study confirmed the presence of an information cascade framework, which holds that investors outweigh other investors’ information compared with their private information and, thus, follow others.

Availability of information reduces the risk in the market. Cognitive attributes of investors are affected by information availability when investors have sufficient knowledge and they decide to invest. Many firms do not give information about their firms, so investors invest by following others who have more experience. Shefrin and Statman (2011) claimed that investors may ignore material information and start following others because of the fear of failure. Investors show herd behavior to preserve their reputation. If the failure occurs on the decision that was made by an individual on his attribution, he will suffer from a high amount of regret; but if the failure occurs on the decision made by many other investors, the amount of regret is low. In an efficient market, investors are rational, and prices of securities will reflect all the available information (Ross et al., 2016). In reality, the situation is different when investors’ decisions depend upon their behaviors and emotions. According to Easley and Kleinberg (2010), investors follow the decisions of others, although they know that others’ decisions may also be wrong instead; there is no solid reason to mitigate the actions of others, but still, they follow others, believing that others might have more relevant information. Similarity, Chaffai and Medhioub (2018) stated that it is hard for investors to logically analyze the new information that is available, and due to this reason, investors avoid the available information and take decision by following other investors in the market.

The illusion of control is a situation in which investors overstated their skills to control events, and they consider that the outcomes of unpleasant events are in their hands (Metilda, 2015). The individual investor who is affected by the illusion of control thinks that his skills are very worthy and productive. He considers himself very skillful and can take decisions in any situation. It is demonstrated that investors having an illusion of control attribution are consider as a more risk-taker in investing (Boyd and Vozikis, 1994). The illusion of control is a bias in which individual investors overestimated their capability to control any events, and they think that the outcomes of unpleasant events are in their control (Metilda, 2015). Fellner (2009) examined the illusion of control bias in the portfolio allocation of individual investment. Her study used z-Tree computerized experiment, which was developed by Fischbacher in 2007. The experiment was conducted in the Max Planck Research Laboratory. The age of participants was from 18 to 32, and their average income is 12.5 $. In line with excessive extrapolation, the higher the number of observed positive prior outcomes, the more likely is a positive prediction and in turn a higher investment. Another study of Ajmal et al. (2011) examined the illusion of control in perceived market efficiency. This study used a questionnaire, and regression model was applied. The results showed that the illusion of control has a significant impact on an investor’s decision making. Handoyo et al. (2020) examined the impact of different behavioral biases (anchoring, loss aversion, illusion of control, and overconfidence) on herding. This study found that behavioral biases have a significant impact on investment decisions, especially for naïve investors.

Self-attribution is described as situation in which an individual attributes success to his skill and failures due to unpleasant events (Hoffmann and Post, 2014). Pompian (2012) explained that self-attribution is a motivational and cognitive component. Generally, an individual gives accomplishment credit to his intrinsic aspects, e.g., ability and knowledge, and blames other external conditions for his failure. The study of Mushinada (2020) concluded that self-attribution and overconfidence could significantly impact investment decisions.

**FIGURE 1:**
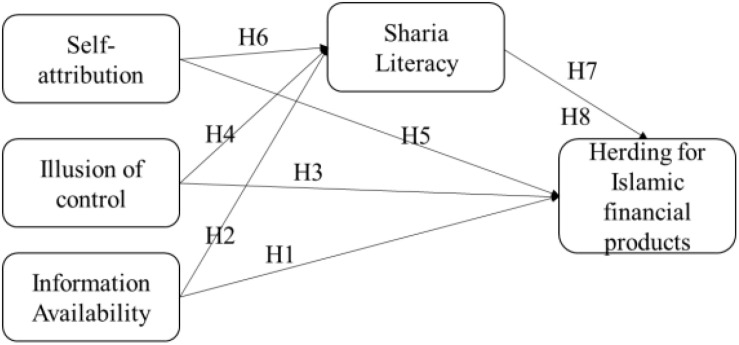
Research framework. H1, self-attribution has a significant impact on herding for Islamic financial products; H2, illusion of control has a significant impact on herding for Islamic financial products; H3, information availability has a significant impact on herding for Islamic financial products; H4, self-attribution has a significant impact on shariah literacy; H5, illusion of control has a significant impact on shariah literacy; H6, information availability has a significant impact on shariah literacy; H7: shariah literacy has a significant impact on herding for Islamic financial products; H8: shariah literacy mediates the relationship between self-attribution, illusion of control, information availability, and herding for Islamic financial products.

Yusuff and Mansor (2016) indicated that decision making of investors is affected by Islamic financial literacy, individual religiosity level, and product knowledge. These are the few factors that influence the individual decision-making process. Islamic financial literacy plays a major role in impelling Muslims to make the right choice in their decision of investment. The Muslims must have information on finance management because it can direct them to make a decent choice that is understandable in the Islamic point of view. A decent choice will assist them be successful in this world and hereafter. Only good understanding of Islamic principles enforces them to educate themselves about Islamic financial literacy and invest in it. Prior studies show that people with a high level of Islamic financial literacy tend to put their assets in Islamic investment. The government is trying to increase the level of Islamic financial literacy and provide additional Quran and Hadith education to put public belief in Islamic principles. It is found that there is a significant relationship between Islamic financial literacy and financial management planning. A person is better in managing his finances if his level of financial literacy is high; and if a person has high Islamic financial literacy, then he prefers Islamic investment (Boon et al., 2011; Arrondel et al., 2013; Agarwal et al., 2015). Widityani et al. (2020) found that the tendency for Islamic products would be higher among investors with high Islamic financial literacy. Similarly, past studies such as Glaser and Weber (2007), Hung et al. (2009), Boon et al. (2011), Arrondel et al. (2013), and Agarwal et al. (2015) argued that information availability along with shariah literacy could have a significant impact on financial decision making and choice of financial products, i.e., conventional or Islamic. For instance, a person with more information availability and higher Islamic financial literacy is more likely to invest in Islamic financial products. Claimed that Islamic financial literacy may produce better financial returns for investors and rationally investment behavior. Based on literature, Figure 1 shows the research framework and hypotheses that may be proposed.

## Methodology

The objective of this study is to examine the impact of self-attribution, illusion of control, information availability, and herding for Islamic financial products with the mediating role of sharia literacy. This study utilized a quantitative research approach to address the study objective. The data for this study were collected through an adoptive questionnaire on a five-point Likert scale. The items for information availability, self-attribution, illusion of control, and herding were adopted from Sabir et al. (2018), while Islamic sharia literacy items were adopted from Albaity and Rahman (2018). The population of the study was composed of investors investing in Islamic financial products and 11 banks that were targeted for data collection, four pure Islamic, and seven conventional banks with Islamic banks.

The principle outlined by Baron and Kenny (1986) was used to justify the mediating role of Islamic sharia literacy. As per Baron and Kenny (1986), a prior relationship between variables, i.e., independent, dependent, and mediating, must exist. Partial least square structural equation modeling (PLS-SEM) is used to test the hypothesis. The PLS-SEM technique is superior to other statistical methods in many ways. For example, it has no sample size restriction, is effective for statistical model building along with forecasting, is precise and accurate in estimation, has soft modeling assumptions, does not require normality of data, and is suitable especially in case of mediation (Iacobucci et al., 2007; Osborne, 2011; Hair et al., 2014, 2017; Ramli and Nartea, 2016). Moreover, SEM is a combination of two powerful statistical approaches, exploratory factor analysis and structural path analysis, which enable the simultaneous assessment of the measurement model and the structural model (Hair et al., 2017).

## Data Analysis

A total of 500 questionnaires were distributed among respondents, 422 respondents returned the filled questionnaires, and 410 were used for the analysis. A researcher like Cain et al. (2017) and Hair et al. (2017) suggested that data need to be checked for multivariate skewness and kurtosis using the software available at^1^. The results showed that the present study data were not multivariate normal; thus, we continued to use the SmartPLS, which is a non-parametric analysis software due to multivariate normality issues. An analysis like descriptive analysis, reliability, convergent validity, discriminant validity, loadings, and path analysis was performed to analyze the collected data.

Table 1 reports descriptive statistics, calculated through SPSS, of the respondent’s profile. The results revealed that 63.2% of respondents were male, and 36.8% of females responded to the questionnaire. Descriptive statistics further reported that the majority of respondents (46.3%) were from 26–35 age bracket followed by 29.8% belonging to 18–25 age bracket. Descriptive statistics also reported that majority of the respondents hold either a graduate or post-graduate degree, thereby enabling them to intelligently respond to the questions.

**TABLE 1:** Respondent’s descriptive profile.

Particulars		Frequency	%
Gender	Male	259	63.2
	Females	151	36.8
Age	18–25	120	29.8
	26–35	190	46.3
	36–45	69	16.8
	46 and above	29	7.10
Marital status	Single	184	44.9
	Married	226	55.1
Qualifications	Graduates	169	41.2
	Post-graduates	179	43.7
	Others	62	15.1

The validity of the constructs was observed by a measurement model with convergent validity and discriminant validity. To observe the convergent validity of the constructs, factor loadings, composite reliability (CR) and average variance extracted (AVE) were used. According to Hair et al. (2014), if the factor loading of items is ≥0.50, CR ≥ 0.80, and AVE ≥ 0.50, then the constructs’ validity is convergent or otherwise. The values for factor loadings were greater than 0.5 as reported in Table 2; hence, the model has good fitness. Moreover, the value of CR for all variables satisfied the criteria except for SA. Similarly, the values of average variance were greater than 0.5, indicating the presence of convergent validity.

**TABLE 2:** Convergent validity.

Construct	Items	Loadings	Composite reliability	Average variance extracted
HB	HB1	0.78	0.80	0.50
	HB2	0.79		
	HB3	0.58		
	HB4	0.64		
IA	IA1	0.67	0.83	0.50
	IA3	0.72		
	IA4	0.75		
	IA5	0.75		
	IA6	0.65		
	IA1	0.67		
IOC	IOC1	0.78	0.84	0.52
	IOC2	0.74		
	IOC3	0.73		
	IOC4	0.70		
	IOC5	0.64		
SA	SA1	0.69	0.78	0.55
	SA2	0.71		
	SA3	0.81		
SL	SL2	0.58	0.84	0.51
	SL4	0.63		
	SL6	0.78		
	SL7	0.78		
	SL8	0.76		

*HB, herding behavior; IA, information availability; IOC, illusion of control; SA, self attribution; SL, shariah Literacy.*

The value of the hetrotrait–monotrait (HTMT) ratio is explained in Table 2. HTMT ratio is the geometric means of heterotrait–heteromethod correlations divided by the means of the monotrait–heteromethod correlations. The HTMT ratio is an effective way to observe the discriminant validity. According to a model is well fitted if the discriminant validity of the HTMT ratio should be less than 0.85. In Table 3, values of all HTMT ratios are less than 0.85, which present the discriminant validity of the model.

**TABLE 3:** HTMT discriminant validity.

HTMT					

	HB	IA	IOC	SA	SL
HB					
IA	0.658				
IOC	0.588	0.677			
SA	0.616	0.627	0.652		
SL	0.519	0.768	0.528	0.592	

**Discriminant validity**					

	**HB**	**IA**	**IOC**	**SA**	**SL**

HB	**0.705**				
IA	0.470	**0.707**			
IOC	0.428	0.515	**0.720**		
SA	0.386	0.419	0.425	**0.739**	
SL	0.388	0.577	0.412	0.404	**0.712**

**HTMT**					

	**HB**	**IA**	**IOC**	**SA**	**SL**
HB					
IA	0.658				
IOC	0.588	0.677			
SA	0.616	0.627	0.652		
SL	0.519	0.768	0.528	0.592	

*HTMT, hetrotrait–monotrait. The bold values indicates that all other values within the same column must be lower than that.*

In the next phase, after the evaluation of the measurement model, we move to the evaluation of the structural model in SmartPLS. For gaining consistent result, we evaluate structure model through SmartPLS bootstrapping. The structural model gives us results through path coefficient, standard error, t-value, p-value, R^2^, Q^2^, and the final decision of a hypothesis (Hair et al., 2014). To get direct and mediating results of variables, the bootstrapping method was applied (Hair et al., 2014; Soto-Acosta et al., 2016).

Table 4 reports factor loading and variance inflation factor (VIF). VIF analysis was performed to check for potential multicollinearity issues. The value of VIF may be used as a general criterion to figure out potential multicollinearity; for instance, VIF equal to or less than one indicates no correlation; a value between one and five shows moderate collection. In contrast, a value higher than five indicates a strong correlation among variables and the possibility of multicollinearity. As all the values reported above for VIF are close to one, it may be presumed that there is no multicollinearity issue.

**TABLE 4:** Direct results.

Hypothesized path	Path coefficient	Standard error (STERR)	*t* value	Decision	*F* size	VIF	R^2^	Q^2^
IA → HB	0.244***	0.078	3.124	Supported	0.048	1.782	0.299	0.140
IOC → HB	0.192***	0.064	2.982	Supported	0.035	1.484		
SA → HB	0.161***	0.058	2.768	Supported	0.027	1.353		
SL → HB	0.103	0.062	1.670	Unsupported	0.009	1.595		
IA → SL	0.451***	0.051	8.837	Supported	0.223	1.457	0.373	0.182
IOC → SL	0.108*	0.053	2.023	Supported	0.013	1.466		
SA → SL	0.169***	0.060	2.808	Supported	0.035	1.307		

VIF, variance inflation factor.***, **, and * indicate significance at 0, 1, and 5%, respectively.

**FIGURE 2:**
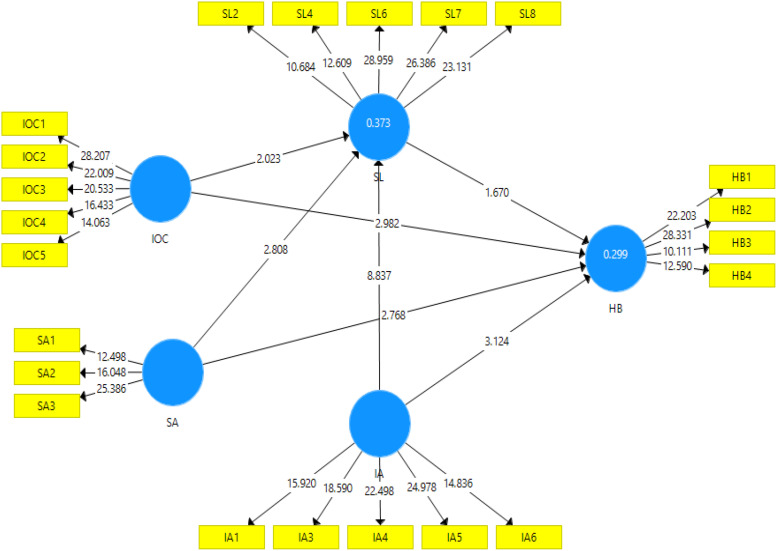
Structural model.

The results for the direct relationship (as reported in Table 4 and Figure 2) reported that all variables, i.e., information availability (β = 0.244, *p* < 0.00), illusion of control (β = 0.192, *p* < 0.00), and self-attribution (β = 0.161, *p* < 0.00), showed a positively significant impact on herding. The results for the direct relationship reported that all variables, i.e., information availability (β = 0.451, *p* < 0.01), illusion of control (β = 0.108, *p* < 0.01), and self-attribution (β = 0.169, *p* < 0.00), showed a positively significant impact on shariah literacy.

## Discussion

This result for herding is in in-line with the findings of Daniel et al. (1998), Fernández et al. (2001, 2011), Nofsinger (2005), Epstein and Schneider (2008), Abreu and Mendes (2012), Nguyen and Schüßler (2012), Stefan and David (2013), Metilda (2015), Mishra and Metilda (2015), Pavlovic (2018), and Narasimha and Mushinada (2018).

According to authors like Keynes (1937), Bikhchandani and Sharma (2000), Chang et al. (2000), Shiller (2003), Chiang and Zheng (2010), Hassairi (2011), and Bashir et al. (2014) market inefficiencies, low self-attribution, and less illusion of control and information cascade framework may lead to herding. In an inefficient market, like Pakistan, investors may believe that either they do not have complete information or others may have better information (information cascade framework), and they may become uncertain and doubtful, which will lead them to follow the herding. Where perfect information does not exist, the feeling of uncertainty leads individuals to think that others are better informed. The greater the investors’ feeling of uncertainty, the more likely they doubt their information. Consequently, investors try to obtain information by observing the financial decision making of others participating in the market.

The results for shariah literacy (β = 0.161, *p* > 0.005) and herding were found to be insignificant, suggesting that shariah literacy may promote rational thinking and independent decision-making behavior among investors rather than inducing them to follow the crowd. This result is in line with the findings of Atkinson and Messy (2012), Setyowati et al. (2018), and Biplob and Abdullah (2019), who also found that higher literacy will encourage people to think and act rationally.

The results presented in Table 5 reported an indirect relationship between information availability, illusion of control, self-attribution, and herding with the mediating impact of shariah literacy through bootstrapping. The previously significant relationships between information availability, illusion of control, self-attribution, and herding turned out to be insignificant with the mediation of shariah literacy. This suggests that shariah literacy could have a significant impact on controlling cognitive biases and encouraging rational investment behavior.

**TABLE 5:** Indirect results.

Hypothesized path	Path coefficient	Standard error (STERR)	*T* value	*p* value	2.50%	97.50%	Decision
IA → SL → HB	0.046	0.029	1.587	0.113	−0.011	0.104	No mediation
IOC → SL → HB	0.011	0.009	1.237	0.216	−0.001	0.035	No mediation
SA → SL → HB	0.017	0.012	1.485	0.137	−0.001	0.046	No mediation

Table 6 shows that Q^2^ values of the items on HB are greater. Eight out of nine items in the endogenous latent variable, i.e., SME performance, are greater than 0. The result deduced that predictive relevance is present in the model. As a conclusion, when a PLS-SEM model exhibits predictive relevance, it will predict well the data points of indicators.

**TABLE 6:** PLS Predictive.

Table predicative:											
		PLS predict						LM		PLS predict—LM	
	RMSE	MAE	MAPE	Q^2^_predict		RMSE	MAE	MAPE	Q^2^_predict	RMSE	Q^2^_predict
HB4	0.86	0.639	23.753	0.118	HB4	0.884	0.660	24.332	0.069	−0.024	0.049
HB2	0.883	0.667	24.609	0.185	HB2	0.899	0.675	24.716	0.157	−0.016	0.028
HB1	1.056	0.816	35.318	0.149	HB1	1.063	0.813	34.891	0.138	−0.007	0.011
HB3	1.04	0.837	35.921	0.082	HB3	1.026	0.820	34.630	0.107	0.014	−0.025
SL4	0.764	0.573	18.710	0.170	SL4	0.748	0.548	17.762	0.203	0.016	−0.033
SL6	0.898	0.695	23.933	0.197	SL6	0.908	0.706	24.462	0.179	−0.010	0.018
SL7	0.736	0.564	17.439	0.191	SL7	0.747	0.571	17.777	0.167	−0.011	0.024
SL8	0.786	0.610	19.524	0.217	SL8	0.799	0.620	19.614	0.191	−0.013	0.026
SL2	0.898	0.702	24.147	0.116	SL2	0.897	0.696	23.610	0.117	0.001	−0.001

*RMSE, root mean square error; MAE, mean absolute error; MAPE, mean absolute percentage error.*

## Conclusion

Due to cognitive biases and low shariah literacy, individual investors follow herd behavior in Islamic Financial Products. Our study acknowledged/documented the influence of cognitive biases information availability, illusion of control, and self-attribution on individual herd behavior in Islamic financial products. Islamic financial literacy is not developed enough, so our study shows the effect of cognitive biases in individual investor decision making. Usually, investors are not financially literate; they are unable to evaluate the good investment products. Most of the investors do not have shariah literacy, or they are not intelligent enough to convert information into knowledge. Thus, in an uncertain situation where information is not fully available, investors indulge in herding behavior because they thought others are better in accessing the good investment decision. Results indicate that cognitive biases information availability, illusion of control, and self-attribution encourage herding behavior in Islamic financial products, but shariah financial literacy mitigates the result of herding behavior in Islamic financial products. The outcome of this study helps us to tell why investors indulge in herding behavior in taking an investment decision. This will also be helpful for investors to predict the different biases in their behavior. This study focuses on the only individuals located in Sialkot, Gujrat, and Gujranwala; and due to time limitation, the sample size is also small. Further research can be done on a large sample size in many different cities to get more general conclusions.

### Practical Implications

The findings of this study have several practical implications for financial managers. As mentioned earlier, herding either positively or negatively would result in financial instability, thereby causing market crashes and investors losing billions of dollars. Shariah literacy would help investors to act rationally, thereby improving market efficiency.

## Data Availability Statement

The raw data supporting the conclusions of this article will be made available by the authors, without undue reservation.

## Ethics Statement

The studies involving human participants were reviewed and approved by Zeeshan Ahmed (Assistant Professor University of Lahore). The patients/participants provided their written informed consent to participate in this study.

## Author Contributions

SD and SM wrote the article. IA collected the data. AS performed the data analysis and discussion. AD and AA-R significantly contributed to address reviewers comments to improve overall write-up of article. All authors contributed to the article and approved the submitted version.

## Conflict of Interest

The authors declare that the research was conducted in the absence of any commercial or financial relationships that could be construed as a potential conflict of interest.

## Footnotes

1 https://webpower.psychstat.org/models/kurtosis
